# Neuralgia of the Testicle

**Published:** 1873-03

**Authors:** 


### NEURALGIA OF THE TESTICLE.

Several years have now elapsed since Dr. Lazarus, of Cuernowitz, reported a number of instances in which spermatorrhœa was accompanied by neuralgia of the testicle. Since then, he has continued to give especial attention to cases of this nature which have come under his notice in his hospital and private practice. He finds that in a very limited number of cases only is the entire testicle affected by neuralgic pains; these are commonly limited to the *epididymis,* more particularly the superior portion, together with a part of the *vas deferens.* The left testicle is more frequently affected than the right. The precise pathological changes which take place in the diseased parts have not yet been satisfactorily made out. In a few instances, however, there has been noticed a moderate swelling of the organ, with slight enlargement of the blood vessels. The predisposing causes of this affection do not differ from those of ordinary neuralgia. It may be the result of idiopathic or traumatic inflammation, of exposure to cold and dampness, or may be induced by the mechanical pressure of some adjacent tumor.

The most common cause, however, of this form of neuralgia is long-continued sexual continence, so that, as would naturally be inferred, the list of this class of sufferers is made up very largely of bachelors. At times, it appears to be a concomitant of approaching impotence, caused here by the engorgement of the blood vessels and seminal ducts. In such cases, marked relief has been known to follow a natural evacuation of these vessels. In other instances, neuralgia of the testicle makes its appearance in the case of broken-down individuals suffering from dyspepsia. Still another cause is the induration which not unfrequently remains behind after a protracted inflammation of the epididymis. In two instances, neuralgia of this organ was found to follow the injection of a strong solution of permanganate of potash and sulphate of copper in the course of a treatment of gonorrhœa. Renal calculi, during their passage along the ureter, may also lead to an attack of this form of neuralgia. In one case seen by Dr. L., the affection was caused by a fall from a lofty scaffolding, by which the lumbar portion of the spinal column received severe injury, resulting in paralysis of the lower extremities and eventual death. Finally, neuralgia of the testicle is not unfrequently an accompaniment of varicocele. The affair generally begins with a sensation of pain in the upper portion of the epididymis, which, continuing without remission, would point to commencing inflammation of that organ, were it not for the absence of the ordinary signs of inflammation, such as redness and swelling. All doubt as to the real nature of the trouble is very soon removed, however, by the change in the severity of the pain, which now comes on in paroxysms of a burning, boring character, accompanied at times by nausea and vomiting. This pain is renewed and aggravated by motion, or whenever any pressure is applied to the scrotum. Cold applications afford no relief whatever to the sufferer; heat, on the other hand, serves to alleviate the pain, particularly when applied in the form of warm baths. This fact will explain why the patient finds himself more comfortable in heated apartments, such as the ball-room or theatre; and also why the paroxysms are less severe in summer than in winter.

Among the agents recommended for the relief of this form of neuralgia, Dr. L. speaks (*Wiener Med. Presse*, July 28, 1872) most highly of sulphate of zinc, given as follows:

℞.—Zinci sulphatis, gran. tria; aquæ distillatæ, unc. quinque; aquæ laurocerasi, drach. unam; Syrup. cort. aurantium, unc. semis. M. Dose, tablespoonful three times daily.

He also advises the daily injection into the posterior wall of the scrotum of a small quantity of the solution of sulphate of zinc, having the strength of one-half grain to the ounce. In obstinate cases it may be necessary to resort to castration.—*Boston Medical and Surgical Journal.*

---

Hemorrhoidal Suppository.—Dr. Henry M. Field, Newton, Massachusetts, (*Journal Gynæcological Society*), recommends this formula for a suppository in hemorrhoidal cases: ℞.—Acid tannic, gr. v.; ext. belladonna, gr. ss-j.; butyr. cacao, q. s.

